# Effect of trace mineral injection on serum trace mineral concentrations and reproductive indicators in sheep synchronised during the anoestrus period

**DOI:** 10.17221/28/2025-VETMED

**Published:** 2025-10-29

**Authors:** Atakan Cortu, Orsan Gungor

**Affiliations:** Department of Veterinary Obstetrics and Gynecology, Faculty of Veterinary Medicine, Burdur Mehmet Akif Ersoy University, Burdur, Turkiye

**Keywords:** ewe, mineral, oestrus, pregnancy, trace element

## Abstract

Sheep production represents an important source of income for farmers in Türkiye; however, reproductive inefficiencies during the anoestrus period pose a major challenge to productivity. This study aimed to evaluate the effects of injectable trace minerals (copper, selenium, manganese, and zinc) on the reproductive performance of Awassi ewes synchronised during the anoestrous period. A total of 200 clinically healthy ewes were randomly allocated to two groups: the trace mineral group (TRACE, *n* = 100), which received 2 ml of a trace mineral solution 14 days before oestrus synchronisation, and the control group (CON, *n* = 100), which received 2 ml of 0.9% NaCl. Oestrus was synchronised using intravaginal sponges containing 60 mg medroxyprogesterone acetate for 12 days, followed by administration of 500 IU equine chorionic gonadotrophin at sponge removal. Ewes were hand-mated for five days following synchronisation. Variables assessed included serum trace mineral concentrations, response to synchronisation, and reproductive outcomes (oestrus rate, pregnancy rate, lambing rate, incidence of multiple births, and dystocia). No significant differences (*P* > 0.05) were found between groups in serum concentrations of copper, zinc, or manganese. Although the duration of oestrus was significantly longer in the TRACE group compared to control (29.66 ± 0.96 h vs 26.09 ± 0.89 h; *P* = 0.006), all other reproductive indicators were similar between groups. These findings suggest that a single pre-synchronisation injection of trace minerals does not significantly improve the reproductive performance or mineral status in anoestrous Awassi ewes. Further research is needed to determine the optimal timing and dosing strategies for supplementing trace minerals in sheep.

Sheep breeding is a vital source of income for farmers, particularly in developing countries such as Türkiye (Gonzalez-Bulnes et al. 2005; Teker 2012). Sheep are reared for milk, wool, meat, manure, and leather, with these products primarily marketed domestically (Gul and Ornek 2019; Martin et al. 2024). However, the high cost of livestock production in Türkiye limits its competitiveness in global markets (Albez 2018). In developed countries, animal husbandry accounts for 60–80% of total agricultural income, whereas in Türkiye, it contributes only 21.68%, indicating that the sector has not yet reached its full national potential (Kan and Direk 2004). Improving reproductive efficiency in sheep could increase the production of animal-derived goods, enhance farmers’ livelihoods, and contribute to national economic growth. Oestrus synchronisation protocols have emerged as a promising method for improving the reproductive performance and productivity of ewes (Hasani et al. 2018).

Trace minerals play a crucial role in supporting reproductive function and overall health in ruminants by influencing key physiological processes, such as follicular development, conception, and embryonic growth (Omur et al. 2016; Stokes et al. 2017; Bhalakiya et al. 2019; Willmore et al. 2021; Gonzalez-Rivas et al. 2023). Copper deficiency, for example, has been linked to reduced pregnancy rates, infertility, sterility, abortion, and retained placenta in multiple species, including rats, pigs, and ruminants (Mudgal et al. 2014; Brasche 2015). Selenium deficiency, which impairs antioxidant capacity, may result in infertility, retained placenta, abortion, and white muscle disease in ruminants (Gabryszuk and Klewiec 2002; Qazi et al. 2018). Manganese deficiency in ruminants has been associated with delayed development of bones and soft tissues, reduced milk yield, infertility, foetal abnormalities, and abortion (McDowell 2003; Studer et al. 2022). Zinc deficiency disrupts the oestrous cycle, potentially preventing ovulation or resulting in the release of degenerated oocytes. It is also associated with increased embryonic and foetal mortality, congenital abnormalities, dystocia, and the birth of weak, non-viable offspring. In sheep specifically, zinc deficiency has been shown to affect embryo implantation, uterine involution, tissue regeneration, and the resumption of the normal reproductive cycles (Apgar et al. 1993; Vickram et al. 2021).

Trace minerals are typically supplied through oral supplementation in the form of mineral blocks, boluses, or liquid premixes (Arthington et al. 2014; Meyer-Binzegger et al. 2022). However, oral administration may result in antagonistic interactions within the gastrointestinal tract, and the process of rumination can further reduce mineral absorption (McDowell 2003). Recent studies have investigated the use of injectable trace minerals administered before oestrus synchronisation in cattle, intending to improve mineral bioavailability and reproductive outcomes (Stokes et al. 2017; Springman et al. 2018; Stokes et al. 2018; Willmore et al. 2021). However, there is limited research on the effects of injectable trace minerals on reproductive performance in sheep (Gonzalez-Rivas et al. 2023). Based on this background, we hypothesised that administering injectable trace minerals to Awassi ewes during the anoestrous period, before oestrus synchronisation, would enhance reproductive performance. Therefore, this study aimed to investigate the effects of a commercial injectable veterinary product (Activate^®^; Alke, Istanbul, Türkiye) containing copper, selenium, manganese, and zinc, on serum mineral concentrations, synchronisation efficacy, and selected reproductive indicators in Awassi sheep.

## MATERIAL AND METHODS

### Animals and experimental design

This study was conducted between April and October 2020 at a sheep farm in the Korkuteli district of Antalya Province and at Burdur Mehmet Akif Ersoy University.

The study population included 200 healthy Awassi ewes, aged 2 to 5 years, with an average body condition score of 3. All ewes had previously given birth and showed no signs of reproductive disorders. Additionally, 20 healthy, experienced Awassi rams were included. Farm records indicated no cases of mineral deficiency or toxicity-related diseases in the flock during the previous two years. The health status of all animals was monitored throughout the study. Fresh water was available *ad libitum.* Apart from the trace mineral injection, no flushing methods or oral mineral supplements were used. During the study, the sheep were fed 350 g of pellet feed (18% crude protein) and 750 g of hay twice daily, in the morning and evening, and grazed on pasture during the day. The vitamin and mineral content of 1 kg of pellet feed is presented in Table 1. Assuming each ewe consumed approximately 1.7 kg of forage from pasture, it was estimated they received about 32.5 mg of copper, 235 mg of manganese, 95.5 mg of zinc, and 0.36 mg of selenium per day, which, according to Ademi et al. (2017), is considered an adequate level of trace elements. The ewes were randomly divided into two equal groups. In the trace mineral group (TRACE, *n* = 100), 2 ml of Activate^®^ was administered intramuscularly 14 days prior to sponge insertion, as specified in the product guidelines. Each 1 ml of Activate^®^ contains 2.5 mg copper gluconate, 1.25 mg sodium selenite, 5 mg manganese gluconate, and 5 mg zinc gluconate. In the control group (CON, *n* = 100), 2 ml of 0.9% NaCl was administered intramuscularly at the same time point.

**Table 1:** Vitamin and mineral content of pellet feed given to sheep (for 1 kg)

Vitamin/mineral type	Value
Vitamin A	5 000 IU
Vitamin D	1 000 IU
Vitamin E	10 mg
Iron	25 mg
Copper	5 mg
Zinc	25 mg
Manganese	25 mg
Sodium	0.36%
Selenium	0.075 mg
Iode	0.4 mg
Cobalt	0.075 mg
Crude protein	14%
Crude oil	3.4%
Crude cellulose	8.84%
Ash	6.10%

IU = International Unit; mg = milligram

### Determination of reproductive indicators

For oestrus synchronisation, each ewe had a minimum interval of two months since its last lambing. An Esponjavet^®^ sponge (Hipra, Girona, Spain) containing 60 mg of medroxyprogesterone acetate was inserted into the vagina using a specialised applicator. The sponges remained in place for 12 days and were removed on the twelfth day. At sponge removal, 500 IU of equine chorionic gonadotrophin [(eCG) Oviser^®^; Hipra, Girona, Spain] was administered intramuscularly to both groups. Subsequently, 20 healthy rams that had been separated from the herd for two months were reintroduced. The sheep were hand-mated for five days, beginning 12 h after sponge removal, between 07:00–09:00 a.m. and 7:00–9:00 p.m., at a ratio of one ram per 10 ewes. Mating times were recorded to determine the number of ewes displaying oestrus. The interval between the eCG administration and the first mating was defined as the time to the onset of oestrus. The oestrus period was considered to have ended when a previously mated ewe refused to mate during the following observation. The time interval between the first and last mating was designated as the total duration of oestrus. At the end of the oestrus monitoring period, the rams and non-oestrous ewes were separated from the herd. Transrectal and transabdominal pregnancy examinations were conducted between 40 and 50 days after mating using ultrasonography (7.5 MHz linear probe; Hasvet^®^ 838; Hasvet, Antalya, Türkiye). Ewes confirmed as pregnant were separated from the non-pregnant ones and maintained in a separate pen until lambing. The number of lambs born per ewe was recorded. Ewes that failed to deliver within one hour of the onset of parturition were classified as experiencing dystocia (Jacobson et al. 2020). The incidence of oestrus, interval from eCG treatment to onset of oestrus, mean oestrus duration, and rates of pregnancy, conception, lambing, singletons, twins, triplets, multiple births, and dystocia were evaluated using data from oestrus monitoring, pregnancy examinations, and lambing records. These indicators were defined according to Koyuncu and Yerlikaya (2007), as shown in Table 2.

**Table 2:** Methods used to determine reproductive indicators

Reproductive indicator	Determination method
Oestrus rate (%)	(number of ewes in estrus/total number of ewes) × 100
Time between eCG and onset of oestrus	time elapsed between eCG injection to the onset of oestrus (hours)
Average oestrus duration	time between the start and end of oestrus (hours)
Pregnancy tate (%)	(number of pregnant ewes/total number of ewes) × 100
Conception rate (%)	(number of pregnant ewes/number of ewes showing oestrus) × 100
Lambing rate (%)	(number of ewes giving birth/total number of ewes) × 100
Single birth rate (%)	(number of ewes giving singleton births/total number of ewes giving birth) × 100
Twin birth rate (%)	(number of ewes giving twin births/total number of ewes giving birth) × 100
Triplet birth rate (%)	(number of ewes giving triplets/total number of ewes giving birth) × 100
Multiple birth rate (%)	(number of ewes giving multiple births/total number of ewes giving birth) × 100
Dystocia rate (%)	(number of dystocia cases/number of ewes giving birth) × 100

% = percent; eCG = equine chorionic gonadotropin

### Determination of trace mineral concentrations

Blood samples were collected from the left or right jugular vein of the 10 ewes per group using a Vacutainer and blood collection holder to determine the levels of copper, manganese, and zinc. Selenium was excluded due to budgetary constraints. Samples were collected three times into sodium heparin tubes (Vacuette^®^; Greiner, Kremsmüster, Austria): 14 days before sponge insertion (pre-treatment), on the day of sponge insertion, and on the day of sponge removal. Sheep were not fed before sampling. Blood samples were centrifuged at 1 500 × *g* for 10 min, and the resulting plasma was stored at –20 °C until analysis. For trace mineral levels, the samples were thawed, and 1 ml of each sample was weighed using a precision scale. Subsequently, 8 ml of 65% nitric acid and 1 ml of 30% hydrogen peroxide were added. The mixture was then heated at 170 °C for 35 min in a START D Microwave Digestion System. After digestion, ultrapure water was added to bring the total volume to 50 ml. The Optima 8000 inductively coupled plasma optical emission spectrometry (ICP-OES) system was activated and optimised. The following wavelengths were used: 324.754 nm for copper, 257.610 nm for manganese, and 213.857 nm for zinc. Calibration was performed using certified standard solutions, and a calibration curve (*R*^2^ > 0.999) was generated for each element. The prepared samples were introduced into the instrument’s sample inlet system. Trace mineral levels were determined via ICP-OES (Douvris et al. 2023).

### Statistical analysis

Descriptive statistics were used to calculate the mean values of trace mineral concentrations before and after treatment within each group. A one-way analysis of variance (ANOVA) was employed to compare trace mineral concentrations both within and between groups. Descriptive statistics were also used to determine the mean values of the time from eCG treatment to the onset of oestrus and the duration of oestrus within groups; a dependent samples *t-*test was applied to assess differences in these data between groups. The chi-square (χ²) test was used to compare reproductive parameters. A significance level of *P* < 0.05 was adopted for all analyses. All data obtained in this study were analysed using Minitab v17 software (Minitab LLC, USA).

## RESULTS

It was observed that the sponges remained in the vagina throughout the 12 days in both the TRACE and CON groups. No animals became ill or were excluded from the study during this period. It was determined that four ewes in each group experienced abortions between days 55 and 82 of gestation.

### Trace mineral concentrations

#### COPPER

Copper concentrations (μg/l) in the TRACE group were measured at the 1^st^, 2^nd^, and 3^rd^ time points as 1 085 ± 103, 1 009.4 ± 99.2, and 1 053.1 ± 53.3, respectively. In the CON group, the corresponding values were 977.6 ± 49.2, 1 096.4 ± 86.8, and 1 159.6 ± 70.6. Statistical analysis revealed no significant differences in copper concentrations across the three time points within either the TRACE or CON group when analysed separately (*P* = 0.832 and *P* = 0.192, respectively). Additionally, no statistically significant differences were observed between the TRACE and CON groups at any of the three measurement points (*P* = 0.491, *P* = 0.745, and *P* = 0.130, respectively).

#### MANGANESE

Manganese concentrations (μg/l) in the TRACE group were measured at the 1^st^, 2^nd^, and 3^rd^ time points as 1.976 ± 0.135, 2.036 ± 0.123, and 1.956 ± 0.129, respectively. In the CON group, the corresponding values were 1.834 ± 0.138, 1.887 ± 0.072, and 1.859 ± 0.080. Statistical analysis revealed no significant differences in manganese concentrations across the three time points within either the TRACE or CON group when analysed separately (*P* = 0.902 and *P* = 0.932, respectively). Similarly, no statistically significant differences were found between the TRACE and CON groups at any of the three measurement points (*P* = 0.591, *P* = 0.508, and *P* = 0.555, respectively).

#### ZINC

Zinc concentrations (μg/l) in the TRACE group were measured at the 1^st^, 2^nd^, and 3^rd^ time points as 674.8 ± 14.9, 696.1 ± 21.4, and 667.9 ± 8.0, respectively. In the CON group, the corresponding values were 673.9 ± 25.8, 635.2 ± 10.0, and 665.9 ± 12.1, respectively. Statistical analysis revealed no significant differences in zinc levels across the three time points within either the TRACE or CON group when analysed separately (*P* = 0.426 and *P* = 0.266, respectively). Likewise, no statistically significant differences were observed between the TRACE and CON groups at any of the three measurement points (*P* = 0.427, *P* = 0.139, and *P* = 0.110, respectively).

### Reproductive indicators

Oestrus onset following eCG treatment occurred at 38.0 ± 0.7 h in the TRACE group and 36.8 ± 0.7 h in the CON group (*P* = 0.181). The mean duration of oestrus was 29.6 ± 0.9 h in the TRACE group and 26.0 ± 0.8 h in the CON group (*P* = 0.006). Oestrus was observed in 87% (87/100) of ewes in the TRACE group and 86% (86/100) in the CON group (*P* = 0.836). The pregnancy rate was 66% (66/100) in the TRACE group and 63% (63/100) in the CON group (*P* = 0.658). The conception rate was 75.8% (66/87) in the TRACE group and 73.2% (63/86) in the CON group (*P* = 0.694). The lambing rate was 62% (62/100) among ewes in the TRACE group and 59% (59/100) among ewes in the CON group (*P* = 0.664). The singleton birth rate was 59.6% (37/62) in TRACE group and 59.3% (35/59) in CON group (*P* = 0.968). Twin, triplet, and multiple birth rates in the TRACE group were 35.4% (22/62), 4.8% (3/62), and 40.3% (25/62), respectively. Corresponding rates in the CON group were 35.5% (21/59), 5.0% (3/59), and 40.6% (24/59), respectively (*P* = 0.990, *P* = 0.950, and *P* = 0.968, respectively). The dystocia rate was 14.5% (9/62) in the TRACE group and 10.1% (6/59) in the CON group. Dystocia occurred more frequently in singleton births in both groups, with rates of 66.6% (6/9) in TRACE and 83.3% (5/6) in CON (*P* = 0.468). The reproductive indicators obtained from both groups are presented in Table 3.

**Table 3:** Reproductive indicators in the TRACE and CON groups

Reproductive indicator	Trace group	Control group
Time between eCG injection and oestrus onset	38.0 ± 0.7 h^a^	36.8 ± 0.7 h^a^
Average oestrus duration	29.6 ± 0.9 h^a^	26 ± 0.8 h^b^
Oestrus rate (%)	87^a^ (87/100)	86^a^ (86/100)
Pregnancy rate (%)	66^a^ (66/100)	63^a^ (63/100)
Conception rate (%)	75.8^a^ (66/87)	73.2^a^ (63/86)
Lambing rate (%)	62^a^ (62/100)	59^a^ (59/100)
Singleton birth rate (%)	59.6^a^ (37/62)	59.3^a^ (35/59)
Twin birth rate (%)	35.4^a^ (22/62)	35.5^a^ (21/59)
Triplet birth rate (%)	4.8^a^ (3/62)	5^a^ (3/59)
Multiple birth rate (%)	40.3^a^ (25/62)	40.6^a^ (24/59)
Total number of lambs	90	86
Dystocia rate (%)	14.5^a^ (9/62)	10.2^a^ (6/59)

^a,b^Different superscript letters in the same row indicate a statistically significant difference (*P* < 0.05)

% = percent; eCG = equine chorionic gonadotropin

## DISCUSSION

As few studies have been conducted specifically in sheep, the findings of this study are compared with research evaluating the effectiveness of injectable or bolus forms of trace minerals in both sheep and cattle. Daugherty et al. (2002) administered 1 ml/150 kg of a trace mineral preparation (Multimin^®^ 90; Multimin USA, Inc., Fort Collins, USA), containing 15 mg copper gluconate, 5 mg sodium selenite, 10 mg manganese gluconate, and 60 mg zinc per ml, along with 6.22 IU/kg of vitamin E (Agripharm^®^, Aspen, USA), to cattle approximately 30 days prior to calving. They reported a significant increase in maternal serum selenium concentrations in the experimental group at calving, with no significant changes in the concentrations of other trace minerals. Nennich et al. (2010) injected Multimin^®^ 90 at a dose of 1 ml per calf (*n* = 60) into 1-day-old calves and found no changes in serum trace mineral concentrations after 43 days. In contrast, some studies have reported changes in serum mineral concentrations following supplementation (Kirchhoff 2012; Stokes et al. 2018). Kirchhoff (2012) observed a statistically significant increase in serum copper, zinc, manganese, and selenium concentrations in bulls (*n* = 26) 8 h after injection with Multimin^®^ 90 at a dose of 1 ml/68 kg; however, these concentrations returned to baseline within 24 hours. Stokes et al. (2018) found that in heifers (*n* = 30) injected with 1 ml/45 kg of Multimin^®^ 90 on days 221, 319, 401, and 521 post-calving, serum copper and selenium concentrations were significantly elevated on day 422 compared to day 221. However, they noted no changes in serum zinc or manganese concentrations. In sheep, Abdollahi et al. (2015) administered a bolus containing 0.396 g iron, 0.333 g copper, 0.036 g zinc, 0.024 g iodine, 0.008 g selenium, 0.06 g cobalt, 0.711% magnesium, 6.9% calcium, and 0.312% sodium to ewes (*n* = 80). The first group received one bolus 4 weeks before CIDR-G + eCG treatment, the second group received two boluses, the third group received CIDR-G + eCG without a bolus, and the fourth group received CIDR-G only. Serum copper and selenium concentrations were significantly higher on the day of mating and in the third month of pregnancy in the bolus groups, whereas the zinc and manganese concentrations remained unaffected.

Hemingway et al. (2001) assigned ewes (*n* = 120) to four equal groups and administered the following: the first group received one bolus containing 4 700 mg zinc, 100 mg iodine, 3 250 mg manganese, 90 mg cobalt, 50 mg selenium, 5 300 mg copper, 268 000 IU vitamin A, 54 000 IU vitamin D3, and 800 IU vitamin E; the second group received an injection of 12.5 mg copper; the third group received 3 400 mg oral copper; and the fourth group received no treatment. By the fourth month of pregnancy, serum copper concentrations in the bolus group had increased significantly compared to pre-treatment concentrations. In the present study, serum copper concentrations exceeded the reference range of 570–760 μg/l, as reported by Herdt and Hoff (2011) and Schweinzer et al. (2017), at all measurement points in both groups, which may be beneficial during mating and pregnancy (Stokes et al. 2017). In contrast, serum zinc concentrations in both groups remained below the reference range of 700–1 400 μg/l throughout the study. Even on the day the sponges were removed, zinc concentrations did not reach the reference range in both groups. Although the reason is unclear, a hepatic deficiency is unlikely, as no clinical symptoms of zinc deficiency, such as loss of appetite, weight loss, itching, or alopecia, were observed (Stokes et al. 2017). However, persistently low serum zinc suggests a subclinical deficiency, which may affect production and reproduction (Whitelaw et al. 1979). Serum manganese concentrations remained within the reference range (1–6 μg/l) in both groups across all time points (Herdt and Hoff 2011). In this study, trace mineral injection administered 14 days before the intravaginal sponges did not significantly alter serum copper (*P* = 0.832), zinc (*P* = 0.426), or manganese (*P* = 0.902) concentrations on the days of sponge insertion and eCG administration. In contrast to Kirchhoff (2012), who measured serum trace mineral concentrations 8 h post-injection, the measurements in this study were taken 14 and 26 days later. The lack of effect may be due to this time gap, or the absence of repeated injections, as used by Stokes et al. (2018).

The half-life of drugs can vary depending on the route of administration (Erkent and Koytchev 2008). Boluses release their contents gradually into the bloodstream over approximately six months, resulting in a longer duration of action. In contrast, injectable trace minerals are absorbed more rapidly and may not maintain an elevated serum trace mineral concentration over time. This may explain why serum mineral concentrations did not increase following injection in the present study, unlike the findings of Abdollahi et al. (2015) and Hemingway et al. (2001).

Achieving a high oestrus rate is critical for the success of synchronisation protocols (Zeleke et al. 2005). However, monitoring oestrus can be challenging in small ruminant herds, especially when synchronisation is applied. Knowing the time to oestrus onset and the average duration of oestrus facilitates more efficient monitoring (Romano 2004). Follicular fluid reflects the trace mineral content of blood and plays a role in oocyte maturation and development (Albomohsen et al. 2011). During the follicular growth phase, trace mineral levels in the follicle increase, likely due to changes in the blood-follicle barrier (Gerard et al. 2002).

In the present study, the time from eCG administration to the oestrus onset was 38.0 ± 0.7 h in the TRACE group and 36.8 ± 0.7 h in the CON group. Although the difference was not statistically significant (*P* = 0.181), the oestrus onset occurred later in the TRACE group. Mean oestrus duration was 29.6 ± 0.9 h in the TRACE group and 26.0 ± 0.8 h in the CON group, with the TRACE group exhibiting a statistically significant increase of 3.5 h (*P* = 0.006). The oestrus rate was 87% in the TRACE group and 86% in the CON group (*P* = 0.836). Although we hypothesised that trace mineral injection might increase the number of ewes entering oestrus, shorten the time to onset, and stimulate follicular development, the findings suggest that a single injection administered 26 days before eCG treatment did not achieve these effects. While the oestrus rate was slightly higher in the TRACE group, the difference was not significant.

Conception and pregnancy rates are crucial for evaluating the success of oestrus synchronisation. Even when the oestrus rate is high, low conception or pregnancy rates can undermine reproductive efficiency (Harl 2014). Most embryo losses occur between days 10 and 21, during maternal recognition of pregnancy, often due to corpus luteum regression (Hostetler et al. 2003). Free radicals can suppress luteinising hormone pulsation and induce luteal cell apoptosis (Al-Gubory et al. 2004). Trace minerals protect oocytes, embryos, and luteal tissue by neutralising oxidative damage (Al-Gubory et al. 2004; Oral et al. 2015). Additionally, injectable trace minerals enhance antioxidant capacity in ruminants (Machado et al. 2014), potentially counteracting oxidative stress caused by progesterone-releasing sponges (Oral et al. 2015). Moreover, trace minerals support the maternal immune system through their immunomodulatory effects, promoting embryo survival during early gestation (Lee et al. 1988; Starkey et al. 1988; Cross and Roberts1989).

Gonzalez-Rivas et al. (2023) reported no significant effect of trace mineral injection on conception and pregnancy rates in ewes (*n* = 1 484) administered Multimin^®^ Plus 30 days prior to joining. Conversely, Kirchhoff (2012) observed a pregnancy rate of 51.2% in cows treated with Multimin^®^ 90 versus 25.5% in untreated controls. Similarly, Sales et al. (2011) reported a pregnancy rate of 43% in heifers (*n* = 375) synchronised with progesterone and injected with 1 ml/50 kg Multimin^®^ 90 25 days before embryo transfer, compared to 30% in the control (*n* = 451).

In this study, the pregnancy rates were 66% and 63%, the conception rates were 75.8% and 73.2%, and the lambing rates were 62% and 59% in the TRACE and CON groups. Although conception, pregnancy, and lambing rates were higher in the TRACE group than in the CON group, these differences were not statistically significant (*P* = 0.694, *P* = 0.658, and *P* = 0.664, respectively). While not statistically significant, the pregnancy rate was numerically higher in ewes injected with trace minerals, which is consistent with findings from studies in cattle. The higher conception, pregnancy, and lambing rates observed in the TRACE group may be attributed to the immunomodulatory effects of trace minerals and their role in enhancing antioxidant concentrations. However, as antioxidant levels were not measured and no immunological function tests were performed in this study, no definitive conclusions can be drawn in this regard.

One advantage of sheep breeding is that most sheep breeds are capable of producing multiple lambs per litter (Wildeus 2000). In ewes, nutritional status and, consequently, the availability of nutrients within the metabolism are directly linked to ovulation rate and litter size (Petrovic et al. 2012). It is known that ewes with higher vitamin and mineral levels exhibit an increased number of FSH-sensitive follicles at the onset of the follicular wave, enabling more antral follicles to be selected and reach ovulatory size (Munoz-Gutierrez et al. 2002).

Abdollahi et al. (2015) reported multiple birth rates of 80% (16/20, *n* = 20) in the group receiving two mineral boluses, 30% (6/20, *n* = 20) in the group receiving one bolus, 33.3% (6/18, *n* = 18) in the CIDR-G + eCG-only group, and 25% (4/16, *n* = 16) in the CIDR-G-only group, noting that the two-bolus group exhibited a statistically significantly higher multiple birth rate. Hemingway et al. (2001) reported twin birth rates of 65.5% (19/29, *n* = 29) in the group administered a bolus containing vitamins and minerals before joining, 44.8% (13/29, *n* = 29) in the group injected with copper, 39.2% (11/28, *n* = 28) in the group receiving copper orally, and 17.8% (5/28, *n* = 28) in the untreated group, stating that the bolus group had a significantly higher twin birth rate.

In this study, the singleton birth rate was 59.6% in the TRACE group (*n* = 62) and 59.3% in the CON group (*n* = 59), while the multiple birth rate was 40.3% in the TRACE group and 40.6% in the CON group. These results, in terms of pregnancy and multiple birth rates, are comparable to those reported by Gonzalez-Rivas et al. (2023). It appears that trace mineral injection administered before synchronisation, outside the breeding season, does not increase the multiple birth rate in ewes.

Although trace minerals are known to have a greater impact on the reproductive system than macrominerals, macrominerals, particularly calcium and phosphorus, are known to influence follicular development and, consequently, fertility (Talukdar et al. 2016). In addition, vitamin A supports embryonic development, vitamin D3 improves fertility rates, and vitamin E exerts antioxidant effects on the embryo (Kalampokas et al. 2014; Mohd Mutalip et al. 2018; Pilz et al. 2018). In this study, unlike those involving bolus administration, the absence of macromineral and vitamin supplementation may have contributed to the lack of an increase in multiple birth rates (Hemingway et al. 2001; Abdollahi et al. 2015).

Births requiring assistance are classified as dystocia (Zaborski et al. 2009). Dystocia is a major cause of perinatal lamb mortality in ewes. It is recognised that ewes giving birth to a single lamb may be at greater risk of dystocia, as singleton lambs tend to be larger (Jacobson et al. 2020). In one study, the dystocia rate was significantly higher in ewes with singleton births (19%) compared to those with multiple births (7.8%) (Scales et al. 1986). In the present study, the dystocia rate was 14.5% in the TRACE group and 10.1% in the CON group. Although this difference was not statistically significant, the higher dystocia rate in the TRACE group may be associated with the slightly greater proportion of singleton births. These findings are consistent with those reported by Scales et al. (1986). In conclusion, this study showed that a single trace mineral injection administered prior to oestrus synchronisation did not reduce the dystocia rate.

The absence of selenium measurements is a limitation of this study. This should be considered in future research to provide a more comprehensive evaluation of trace mineral status.

In conclusion, this study investigated the effects of trace mineral injection administered before oestrus synchronisation on reproductive performance and trace mineral status in Awassi ewes during the anoestrous period. The findings indicate that, although trace mineral injection did not significantly alter serum copper, zinc, or manganese concentrations, it was associated with numerically higher conception (75.8% vs 73.2%), pregnancy (66% vs 63%), and lambing (62% vs 59%) rates in the TRACE group compared to the CON group but the differences were not statistically significant (*P* > 0.05). Oestrus duration was significantly longer in the TRACE group (29.6 ± 0.96 h vs 26.1 ± 0.89 h, *P* = 0.006), but the time to oestrus onset and oestrus rate remained unaffected. Multiple birth rates (40.3% vs 40.6%) and dystocia rates (14.5% vs 10.2%) also showed no significant differences between groups, indicating that injectable trace mineral supplementation did not improve litter size or reduce birthing difficulties. These findings align with previous studies indicating short-term impacts of injectable trace minerals on reproductive outcomes when administered outside the breeding season. The lack of significant effects may be attributed to the timing of injection, the absence of repeated doses, or the exclusion of macrominerals and vitamins, which are known to influence follicular development and fertility. Further research is warranted to explore the optimal timing, dosage, and combination of nutritional supplements to maximise reproductive efficiency in sheep.
